# Pathogenic evaluation of synonymous *COL4A5* variants in X‐linked Alport syndrome using a minigene assay

**DOI:** 10.1002/mgg3.1342

**Published:** 2020-06-16

**Authors:** Tomoko Horinouchi, Tomohiko Yamamura, Shogo Minamikawa, China Nagano, Nana Sakakibara, Koichi Nakanishi, Yuko Shima, Naoya Morisada, Shinya Ishiko, Yuya Aoto, Hiroaki Nagase, Hiroki Takeda, Rini Rossanti, Shingo Ishimori, Hiroshi Kaito, Masafumi Matsuo, Kazumoto Iijima, Kandai Nozu

**Affiliations:** ^1^ Department of Pediatrics Kobe University Graduate School of Medicine Kobe Japan; ^2^ Department of Child Health and Welfare (Pediatrics) Graduate School of Medicine University of the Ryukyus Nishihara Japan; ^3^ Department of Pediatrics Wakayama Medical University Wakayama Japan; ^4^ Department of Physical Therapy Faculty of Rehabilitation Kobe Gakuin University Kobe Japan

**Keywords:** aberrant splicing, mild phenotype, splicing assay, synonymous variant, X‐linked Alport syndrome

## Abstract

**Background:**

X‐linked Alport syndrome (XLAS) is a progressive, hereditary glomerular nephritis of variable severity caused by pathogenic *COL4A5* variants. Currently, genetic testing is widely used for diagnosing XLAS; however, determining the pathogenicity of variants detected by such analyses can be difficult. Intronic variants or synonymous variants may cause inherited diseases by inducing aberrant splicing. Transcript analysis is necessary to confirm the pathogenicity of such variants, but it is sometimes difficult to extract mRNA directly from patient specimens.

**Methods:**

In this study, we conducted in vitro splicing analysis using a hybrid minigene assay and specimens from three XLAS patients with synonymous variants causing aberrant splicing, including previously reported pathogenic mutations in the same codon. The variants were c.876 A>T (p.Gly292=), c.2358 A>G (p.Pro786=), and c.3906 A>G (p.Gln1302=).

**Results:**

The results from our hybrid minigene assay were sufficient to predict splicing abnormalities; c.876 A>T cause 17‐bp del and 35‐bp del, c.2358 A>G cause exon 29 skipping, c.3906 A>G cause exon 42 skipping, which are very likely to cause pathogenicity. Further, patients carrying c.2358 A>G exhibited a mild phenotype that may have been associated with the presence of both normal and abnormally spliced transcripts.

**Conclusion:**

The minigene system was shown to be a sensitive assay and a useful tool for investigating the pathogenicity of synonymous variants.

## Graphical abstract

The minigene system was shown to be a sensitive assay and a useful tool for investigating the pathogenicity of synonymous variants. Patients carrying synonymous variants can exhibit a mild phenotype that may have been associated with the presence of both normal and abnormally spliced transcripts.

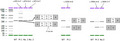

## 1 INTRODUCTION

*COL4A5* (MIM #303630) is a monogenic causative gene of X‐linked Alport syndrome (XLAS; MIM #301050), which may cause end‐stage renal disease accompanied by sensorineural hearing loss and ocular abnormalities (Barker et al., 1990; Kashtan, 1998). *COL4A5* encodes the collagen α5(IV) which constitute the glomerular basement membrane (GBM), cochlea basement membrane, base of the ocular lens, Bowman's capsule, and skin basement membrane. *COL4A5* mutations result in abnormal α5(IV) expression, with a typically complete absence of α5(IV) in men and a mosaic expression pattern in women (Nozu et al., 2019). Males carrying hemizygous mutations exhibit more severe phenotypes than that of females with the genotype‐phenotype correlation in males with XLAS being well established (Bekheirnia et al., 2010; Jais et al., 2000). For instance, truncating mutations result in more severe phenotypes compared with that of non‐truncating mutations, such as missense mutations (Bekheirnia et al., 2010; Jais et al., 2000). We previously reported that *COL4A5* transcripts could also affect the severity of disease by aberrant mRNA splicing resulting in truncation or non‐truncation of the gene product (Horinouchi et al., 2018). Recently, collateral with the progression of genetic analysis technology such as comprehensive sequencing analysis using next‐generation sequencing, the opportunity of genetic diagnosis has been rapidly increasing for cases in which XLAS is suspected, including clinically mild cases. However, a major problem is that many genomic DNA (gDNA) substitutions of unknown pathogenicity may be detected and their pathogenicity must be assessed. In particular, when intronic or synonymous variants are detected, many of them are known to be nonpathogenic; however, it is important to consider the potential effect of splicing. This leads to additional effort and cost that are needed to identify the pathogenic variants that actually cause the aberrant splicing among many non‐pathogenic variants. We believe that variants with high allele frequencies are not likely to be pathogenic. Therefore, only synonymous variants with low allele frequencies (MAF < 0.1%) were evaluated for pathogenicity in this study.

Transcriptional analysis is often difficult to perform as transcripts in the tissues may not be stable. In addition, mRNA extracted from kidney is difficult to obtain in most cases. With respect to *COL4A5*, peripheral blood leukocytes express the transcripts and have been used as an alternative source for transcript analysis. However, it is still sometimes difficult to extract mRNA of sufficient quantity and quality for successful analysis. Amplification of *COL4A5* is also difficult when carrying the mutations that affect the C‐terminus or the N‐terminus, which does not produce the mRNA itself, or a mutation that cause mRNA disruption by a nonsense‐mediated decay. Some groups, including ours, have reported the utility of minigene assays for predicting aberrant splicing in patients with Alport syndrome (Chiereghin et al., 2017; Horinouchi et al., 2018; Malone, Funk, Alhamad, & Miner, 2017). However, there remain insufficient data to determine whether the minigene assay can serve as an alternative to analysis of patient mRNA. Furthermore, determination of the pathogenicity of synonymous *COL4A5* variants has currently not been reported. Here, we report on the evaluation of pathogenicity of synonymous *COL4A5* variants detected in three patients with XLAS using minigene assays.

## 2 MATERIALS AND METHODS

### 2.1 Patients

All procedures involving human participants in this study were performed in accordance with the ethical standards of the Institutional Review Board of Kobe University Graduate School of Medicine and consistent with the 1964 Helsinki Declaration and its later amendments or comparable ethical standards. Informed consent was obtained from all participants included in the study or from their parents or legal guardians.

Patient 1, male, was 19 years old at the time of genetic analysis. Microhematuria and proteinuria had been noted for the patient from 3 years of age. Histological evaluation of a kidney biopsy at 6 years of age revealed lamellation of the GBM and negative expression of α3/α4/α5 (IV). The patient's disease developed into end‐stage renal disease (ESRD) at the age of 18 years. The patient's older brother had also been diagnosed with hematuria and proteinuria at the age of 2 and had an estimated glomerular filtration rate (eGFR) of 32.4 mL/min/1.73 m^2^ at the age of 23. Their mother had hypertension, microhematuria, and proteinuria (0.2 g/g Cr) and her eGFR was 60.7 mL/min/1.73 m^2^ at 49 years of age. None of the family members had ocular abnormalities or hearing loss. The clinical course and gene test results of this family have been previously reported by our group (Fu et al., 2016).

Patient 2, male, was 43 years old at the time of genetic analysis. He had been diagnosed with hematuria from childhood and proteinuria at 33 years of age. His condition developed into ESRD by the time of diagnosis. Evaluation of a kidney biopsy revealed a thin basement membrane, but did not show lamellation. Expression of α5 (IV) was positive. This patient's younger brother had a similar clinical course with hematuria from childhood, proteinuria from 20 years of age, and an eGFR of 32 mL/min/1.73 m^2^ at 31 years of age and their mother had hematuria. The brothers had neither ocular abnormalities nor hearing loss. The clinical courses of siblings suggested that this family may have had relatively mild phenotypes for male XLAS.

Patient 3, female, was 56 years old at the time of genetic analysis. She had microhematuria and occasional proteinuria from 12 years of age. At the age of 30, she presented with gross hematuria with tonsillitis. At 55, a kidney biopsy was performed due to elevated serum creatinine levels. Histological analysis of the biopsy revealed a thin basement membrane and mosaic pattern of α5 expression. At the time genetic testing was performed, her serum creatinine had risen to 1.34 mg/dL, and her eGFR was 32.8 mL/min/1.73 m^2^. She was diagnosed with myopia, but not with other eye disorders or hearing loss. Her mother and younger brother and his daughter had hematuria, but their genetic testing has not been performed, and the detailed clinical course was unknown.

### 2.2 gDNA analysis

Total gDNA was isolated from patients’ peripheral blood leukocytes using a QuickGene Mini‐80 System (Kurabo Industries Ltd.) according to the manufacturer's instructions. For Patient 1, we performed conventional direct sequencing of all exons and exon‐intron boundaries in *COL4A5* (NM: 000495.4) using the Sanger method. For Patients 2 and 3, targeted next‐generation sequencing was performed as described previously using a custom disease panel that included *COL4A3, COL4A4*, and *COL4A5* (Hashimura et al., 2014; Horinouchi et al., 2018; Nozu et al., 2014).

### 2.3 mRNA analysis

For reverse transcription polymerase chain reaction (RT‐PCR) amplification of mRNA and direct sequencing, total RNA was isolated from peripheral leukocytes using RNAlater RNA Stabilization Reagent (Qiagen Inc.) and the RNA was then reverse transcribed into complementary DNA (cDNA) using an EcoDry Kit (Clontech Laboratories, Inc.). RNA from urine sediments were isolated as previously described (Fu et al., 2016; Kaito et al., 2007).

### 2.4 Minigene splicing assays

Hybrid minigene constructs were created in the H492 vector previously developed (Figure S1), which is based on the pcDNA 3.0 Mammalian Expression Vector (Invitrogen; Nozu et al., 2009). We cloned DNA fragments containing exons and introns around the target *COL4A5* variants from the peripheral leukocytes of the three patients and wild‐type controls using In‐Fusion Cloning methods and an HD Cloning Kit (Takara Bio Inc.). To generate the insert fragments, PCR reactions in a 40 μL volume included 100–300 ng of template human gDNA, 20 μL of 2 × Gflex PCR Buffer, 0.25 μmol/L of each primer, and Tks Gflex DNA Polymerase (Takara). The thermal cycling profile included 1 minutes at 94°C, followed by 35 cycles at 98°C for 10 seconds, 60°C for 15 seconds, and 68°C for 30–60 seconds. The inserted sequences are shown in Figure S2. The cloning was done in accordance with the manufacturer's instructions.

To create the constructs used for examining aberrant splicing caused by mutations at the same codon, we cloned wild‐type gDNA and then, introduced mutations by site‐directed mutagenesis using a PrimeSTAR Mutagenesis Basal Kit (Takara Bio Inc.), in accordance with the manufacturer's instructions. The primers used are shown in Table S1.

The hybrid minigenes were confirmed by sequencing before transfection into HEK293T cells using Lipofectamine^®^ 2000 (Thermo Fisher Scientific). HEK293T cells were obtained from the Riken Bio Resource Center Cell Bank (Tsukuba). Total RNA was extracted from cells after 24 hours using an RNeasy^®^ Plus Mini Kit (Qiagen GmbH). An aliquot of total RNA (1 μg) was reverse‐transcribed using RNA to cDNA EcoDry Premix (Double Primed) (Takara Bio Inc.). PCR amplification was performed using a forward primer corresponding to a segment upstream of exon A and a reverse primer complementary to a segment downstream of exon B, as previously described (Nakanishi et al., 2019). PCR products were analyzed by electrophoresis on an Agilent 2100 Bioanalyzer using an Agilent DNA1000 Kit (Agilent Technologies), then directly sequenced.

### 2.5 In silico analysis

The splice sites of each variant were predicted using Human Splicing Finder (http://www.umd.be/HSF3/).

## 3 RESULTS

### 3.1 Synonymous variants detected in our study

**FIGURE 1:**
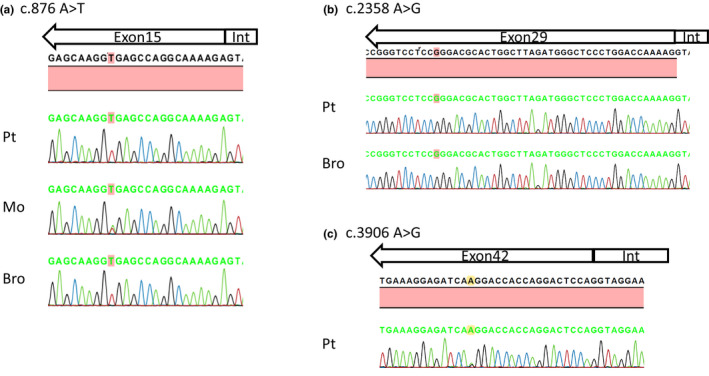
Nucleotide changes in *COL4A5* in patients with X‐linked Alport syndrome. (a) Patient 1 and his brother carried the hemizygous mutation and their mother carried the heterozygous mutation c.876A>T, 16 bp upstream of the exon 15 splice donor site. (b) Patient 2 and his brother carried the hemizygous mutation c.2358 A>G, 38 bp upstream of the exon 29 splice donor site. (c) Patient 3 carried the heterozygous mutation c.3906 A>G, 19 bp upstream of the exon 42 splice donor site. Pt, patient; Mo, mother; Bro, brother; Int, intron

Patient 1 was hemizygous for a c.876 A>T (p.Gly292=) mutation in exon 15, as previously reported (Fu et al., 2016). Single nucleotide substitutions c.2358 A>G (p.Pro786=) in exon 29 and c.3906 A>G (p.Gln1302=) in exon 42 were detected in Patient 2 and Patient 3, respectively (Table 1; Figure 1). No other pathogenic variants were detected in the three patients.

**TABLE 1:** Patients analyzed in this study and their characteristics

Patient	Age (Year)	Sex	Renal function	Kidney biopsy	gDNA substitution	mRNA analysis of patient samples	In vitro minigene assay
Patient 1 (A178)	23	Male	eGFR = 32.4 mL/min/1.73 m^2^	Lamellation of the GBM Negative expression of α5 (IV)	c.876 A>T p.Gly292=	r.[875_891del, 857_891del]	r.[=, 875_891del, 857_891del]
No.1 (Barker et al.)^a^	N/A	Female	ESRD+ (Male patients in family)	N/A	c.874 G>C p.Gly292Arg	N/A	r.=
No.2 (Heiskari et al.)^b^	N/A	Male	ESRD at 15 year	MPGN‐like (light microscopy only)	c.875 G>T p.Gly292Val	N/A	r.=
Patient 2 (A467)	43	Male	ESRD at 43 year	Positive expression of α5 (IV) Thin basement membrane	c.2358 A>G p.Pro786=	r.[=, 2245_2395del]	r.[=, 2245_2395del]
Patient 3 (A514)	56	Female	eGFR = 32.8 mL/min/1.73 m^2^	Mosaic pattern of α5 (IV) expression Thin basement membrane	c.3906 A>G p.Gln1302=	N/A	r.[=, 3791_3924del]
No.3 (Cheong et al.)^c^	N/A	N/A	ESRD as juvenile	N/A	c.3904 C>T p.Gln1302Term	N/A	r.[=, 3791_3924del]

Abbreviation: N/A, not available.

^a^ Barker DF, Denison JC, Atkin CL, Gregory MC. Am J Med Gen. 2001;98:148–60.

^b^ Heiskari N, Zhang X, Zhou J, Leinonen A, Barker D, Gregory M, et al. J Am Soc Nephrol. 1996;7:702–9.

^c^ Cheong HI, Park HW, Ha IS, Choi Y. Pediatr Nephrol (Berlin, Germany). 2000;14:117–21.

### 3.2 mRNA analysis of patient samples

**FIGURE 2:**
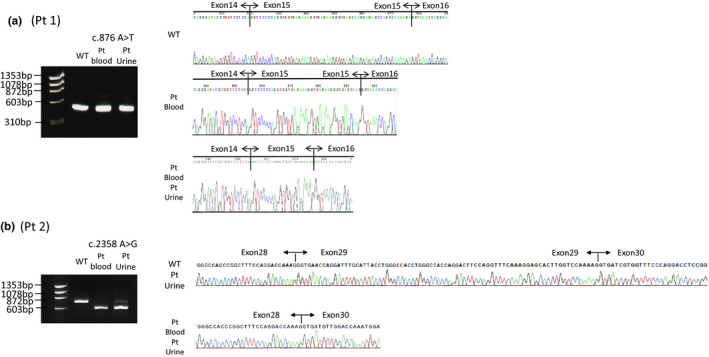
mRNA analysis of specimens from patients with X‐linked Alport syndrome. (a) An isoform with 17‐bp deletion was detected from the blood sample of Patient 1 and a 35‐bp isoform deletion was detected from blood and urine samples. (b) An isoform exhibiting exon 29 skipping was detected in the blood sample from Patient 2 while a small amount of canonical isoform was also detected in the urine sample. Pt, patient; WT, wild‐type

For Patient 1, two variant transcripts were detected with deletions of 35‐ and 17‐bp c.876 A>T r.[857_891del, 875_891del]. We had previously reported only a single transcript with a 35‐bp deletion relative to wild‐type transcripts (Fu et al., 2016). For the current analysis, we resequenced the PCR products to identify the deletions (Figure 2).

For Patient 2, transcripts skipping exon 29 were detected in both the urine and peripheral blood leukocytes (Figure 2). In addition to the truncated transcripts, a small amount of normally spliced transcript was detected in the urine (Figure 2) (c.2358 A>G r.[=, 2245_2395del]). For Patient 3, *COL4A5* transcripts could not be amplified for unknown reasons and in vivo mRNA analysis results were not available.

### 3.3 In vitro splicing

Assays using the minigene system were performed using gDNA fragments from all three patients. In addition, three previously reported pathogenic variants at the same codon (two for Patient 1 and one for Patient 3; Barker, Denison, Atkin, & Gregory, 2001; Cheong, Park, Ha, & Choi, 2000; Heiskari et al., 1996) were analyzed using the minigene system (Table 1).

**FIGURE 3:**
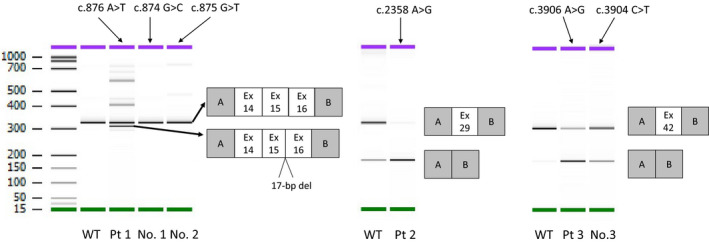
Reverse transcription polymerase chain reaction amplified products of minigene transcripts. Patient 1 (Pt 1) expressed transcripts with a 17‐bp deletion, canonical transcripts, and multiple thin bands that could not be sequenced. Variant No. 1 and No. 2 expressed only canonical transcripts. Patient 2 (Pt 2) expressed mainly transcripts exhibiting exon 29 skipping and a few full‐length transcripts. Patient 3 (Pt 3) expressed transcripts exhibiting exon 42 skipping, which was most likely to occur in the synonymous mutation (Pt 3), followed by the nonsense mutation (No. 3), then the control sequence (WT), and least likely to occur in the full‐length transcript. Pt, patient; WT, wild‐type

For Patient 1 and reported variants (No. 1 and No. 2, respectively), we cloned introns 13 to 16 of *COL4A5*. For Patient 2, we cloned *COL4A5* introns 28 and 29. For Patient 3 and one reported variant (No. 3), we cloned *COL4A5* introns 41 and 42. Patient 1's minigene expressed both full‐length and 17‐bp deletion transcripts, as well as a possible 35‐bp deletion transcript (c.876 A>T r.[=, 875_891del, 857_8921del). The two reported missense variants (Nos. 1 and 2) produced only full‐length transcripts (c.874 G>C r.=, c.875 G>T r=). Patient 2’s minigene primarily expressed a transcript that skipped exon 29, but a few canonical transcripts were also detected (c.2358 A>G r.[=, 2245_2395del]). Patient 3’s minigene expressed more transcripts that skipped exon 42 than did a wild‐type control minigene (c.3906 A>G r.[=, 3791_3924del]). A minigene from a patient with a nonsense mutation reported by Cheong et al. (Cheong et al., 2000) (No. 3) also expressed a transcript that skipped exon 42, which was expressed at a level higher than normal, but lower than that of Patient 3 (c. 3904 C>T r.[=, 3791_3924del]; Figure 3; Figure S3).

### 3.4 In silico splicing assay

The donor site scores for variants No. 1 (c.874 G>C), No. 2 (c.875 G>T), and Patient 1 (c.876 A>T) are shown in Figure S4. The synonymous mutation c.876 A>T created a novel donor site (MaxEnt score 9.6) while neither variant No. 1 nor No. 2 exhibited this novel donor site. The original donor site MaxEnt score was 9.35. Therefore, the novel donor site was stronger than the original. The donor site for the 35‐bp deletion observed in vivo also showed a relatively high score (8.68). For Patient 2 (c.2358 A>G) and Patient 3 (c.3906 A>G), both synonymous mutations failed to alter donor site scores. Furthermore, no novel donor sites were created.

In Patients 2 and 3, alteration of an exonic splicing enhancer site and potential alteration of splicing were demonstrated by the Human Splicing Finder. However, it was difficult to determine which motifs are directly related, because binding affinities for multiple splicing enhancers and silencers can change. This may reflect the fact that these programs are primarily used for screening and are more difficult to use to support functional analysis. Interestingly, the possibility that nonsense mutation c.3904 C>T p.Gln1302Term, in the same codon as variant No. 3, results in aberrant splicing has previously been reported based on extensive in silico analysis (Xiong et al., 2015).

## 4 DISCUSSION

This is the first report evaluating the pathogenicity of synonymous variants in *COL4A5* using an in vitro splicing assay. Our data show that this minigene system works well for the determination of the impact of variants on splicing.

Patient 1 presented with typical Alport syndrome and was considered to be a relatively severe case. Only transcripts with 17‐ or 35‐bp deletions were identified in the urine and peripheral blood from this patient, both of which were out of frame. In addition to canonical transcripts and several other nonspecific bands, in vitro analysis using the minigene system identified transcripts that appeared to contain a 17‐bp deletion and possibly a 35‐bp deletion. The reason for this discrepancy depends on cell type‐specific alternative splicing and the conditions at the time of mRNA extraction; for example, the destruction of mRNA by nonsense‐mediated decay could cause the inconsistency (Acedo et al., 2015). As shown by in silico analysis, this variability may have been due to the mutation creating a novel donor site whose score was higher than that of the original donor site. Moreover, two mutations reported as missense mutations within the same codon failed to induce aberrant splicing in vitro. In silico analysis showed no significant new splice donor sites. This is consistent with these two mutations being simple missense mutations. The detection of normally spliced transcripts from the patient's variant minigene may be a limitation of this in vitro splicing assay.

Patient 2 had a relatively mild case of Alport syndrome, even though he had a *COL4A5* transcript that skipped exon 29 (151 bp), which also resulted in a frameshift. The reason for only mild presentation of disease symptoms in this patient may have been due to the small amount of full‐length transcript still being transcribed/spliced, as detected in the urine sediments.

By the in vitro splicing assay, Patient 3 expressed transcripts that skipped exon 42 (134 bp), but direct mRNA analysis could not be performed on the specimens collected from this patient. It is not uncommon to encounter such a situation in clinical practice. In such cases, if gDNA testing reveals only synonymous variants, a genetic diagnosis of Alport syndrome cannot be made using the usual methods. It is also an inevitable limitation that when analyzing exons, we must consider the possibility that deep intronic mutations could change splicing. In the case of Patient 3, a nonsense mutation in the same codon (c.3904 C>T p.Gln1302Term) has been reported to be associated with a splicing alteration (Xiong et al., 2015). Although we were not able to perform direct mRNA analysis on the patient specimens, our minigene assay revealed that exon 42 skipping was more likely to occur in the order of synonymous mutation, nonsense mutation, and wild type. Based on the previous report and the current results of our minigene assay from this study, we conclude that this synonymous variant should be considered to be causative for Alport syndrome.

In the current study, we determined that our minigene approach was applicable for detecting and analyzing synonymous and missense mutations in *COL4A5*. It may also be useful for predicting splicing stability. However, an important limitation of this minigene system is that it showed exon skipping even with the WT control sequence (Figure 3, WT for Pt 2 and 3). This system overexpresses mRNA synthesis and is likely too sensitive, so exon skipping is observed even with WT control constructs. Therefore, as specimens can only be compared and contrasted with control sequences, it may be difficult to predict the extent of phenotypic rescue due to the presence of a canonical transcript. Considering that transcripts are often unstable and difficult to obtain from patients in sufficient quantity and quality and that alternative splicing may occur in different organs, this minigene system, which can reproduce the splicing patterns of RNA samples from patients that carry spliceogenic variants, may prove very useful as previously described (Acedo et al., 2015; Fraile‐Bethencourt et al., 2017; Fraile‐Bethencourt et al., 2019). We believe that in vitro splicing assays using the minigene system will become more practical in the future.

Recently, a wide spectrum of collagen IV‐related renal disease has attracted attention and the concept of Alport syndrome is changing (Kashtan et al., 2018). With technical advances in genetic analysis, *COL4A5* has also been increasingly identified in patients with chronic kidney disease (Cameron‐Christie et al., 2019; Gast et al., 2016). In addition, like *COL4A5* coding type IV collagen, *COL4A3* traditionally causes autosomal dominant Alport syndrome and is reported to change susceptibility to diabetic kidney disease (Miner, 2019; Salem et al., 2019). We showed that splicing abnormalities may cause severe disease, whereas the presence of full‐length transcripts may attenuate the effect, resulting in milder disease. It is important to recognize splicing alterations as one mechanism that may contribute to the development of “mild” disease.

In conclusion, we presented three cases in which synonymous variants resulted in abnormal splicing leading to the development of disease. The disease severity varied and was milder when normal *COL4A5* transcripts persisted. Furthermore, mild cases may have different clinical courses from those seen for typical Alport syndrome. The minigene system described was shown to be a sensitive and useful tool for investigating whether synonymous variants cause splicing abnormalities.

## CONFLICT OF INTEREST

Kazumoto Iijima has received grant support from Daiichi Sankyo Co., Ltd., and consulting fees from Takeda Pharmaceutical Co., Ono Pharmaceutical Co. Ltd., Boehringer Ingelheim, Astellas Pharma Inc., and Kyowa Kirin Co., Ltd. Kandai Nozu has received lecture fees from Novartis Pharmaceuticals Corporation and consulting fees from Kyowa Kirin Co., Ltd. Kazumoto Iijima and Kandai Nozu have filed a patent application on the development of antisense nucleotides for exon skipping therapy in Alport syndrome.

## AUTHOR CONTRIBUTIONS

T.H. designed the study concept and wrote the manuscript. Ka.N. interpreted the data and wrote the manuscript. T.Y., S.M., C.N., N.S., N.M., Shinya.I., Y.A., and R.R. established and conducted molecular analysis and interpreted the data. M.M. established the minigene assay. Ko.N., Y.S., H.N., H.T., Shingo.I., H.K., and K.I. critically reviewed the manuscript. All authors read and approved the final version of the manuscript.

## Supporting information

Supplementary Material

## Data Availability

The datasets generated and/or analyzed during the current study are available from the corresponding author on reasonable request.
