# Supplementary material for: Pathogenic evaluation of synonymous COL4A5 variants in X‐linked Alport syndrome using a minigene assay
Source: Mol Genet Genomic Med. 2020 Jun 16;8(8):e1342. doi: 10.1002/mgg3.1342 (PMC7434753; doi:10.1002/mgg3.1342)
Supplement: Supplementary file 1 — Supplementary Material [file MGG3-8-e1342-s001.docx]

**Supplementary Fig. 1**

**Supplementary Fig. 2**

**Supplementary Fig. 3**

**Supplementary Fig. 4**

**Supplementary Fig. 1** Schematic of the hybrid minigene. The H492 vector has two cassette exons, A and B, and the insert was cloned between them. The H492 vector also has a cytomegalovirus (CMV) enhancer-promotor and a bovine growth hormone gene (BGH) polyadenylation site.

**Supplementary Fig. 2** Inserted sequences of Patient 1 (c.876 A>T), Patient 2(c.2358 A>G), Patient 3(c.3906 A>G). . For Patient 1 and two reported variants (No. 1; c.874 G>C and No. 2; c.875 G>T), we cloned introns 13-16 of *COL4A5*. For Patient 2, we cloned *COL4A5* introns 28 and 29. For Patient 3 and one previously reported variant (No. 3; c.3904 C>T), we cloned *COL4A5* introns 41 and 42.

**Supplementary Fig. 3** Direct sequencing for minigene transcript analysis. (a) Full-length transcript detected in WT, Pt 1, No. 1, and No. 2. (b) Transcript with a 17-bp deletion identified in Patient 1. (c) Full-length transcript identified in WT. (d) Transcript directly connected to exon A and exon B identified in WT, Pt 2, Pt 3, and No. 3. (e) Full-length transcript identified in WT, Pt 3, and No. 3. Pt, patient; WT, wild-type; Ex, exon.

**Supplementary Fig. 4** Donor site scores (MaxEnt) for Patient 1, No. 1, and No. 2. The original donor site score (right) was 9.35. The upstream novel donor site score was 8.68 (left), which is relatively high, suggesting that it may work as donor site, producing a 35-bp deletion. The synonymous mutation c.876A>T (Pt 1) created a novel donor site (middle), score 9.60, which is stronger than that of the original donor site and induced a 17-bp deletion. Neither No. 1 nor No. 2 create a novel donor site. Pt, patient; WT, wild-type; Int, intron.

**Supplementary Table 1**

Primers used for cloning

Pt 1, No. 1, No. 2 Vector F: 5ʹ-ATCCGGATCCTGGTTTGA-3ʹ

Pt 1, No. 1, No.2 Vector R: 5ʹ-ATCGATGTTAACGCTAGC-3ʹ

Pt 1, No. 1, No. 2 Fragment F: 5ʹ-AGCGTTAACATCGATATCCCATCAGCCAGGTAG-3ʹ

Pt 1, No .1, No. 2 Fragment R: 5ʹ-AACCAGGATCCGGATGGGGATGAATACCCCATTC-3ʹ

Pt 2 Vector F: 5ʹ-ATCCGGATCCTGGTTTGA-3ʹ

Pt 2 Vector R: 5ʹ-ATCGATGTTAACGCTAGC-3ʹ

Pt 2 Fragment F: 5ʹ-AGCGTTAACATCGATCCCCCATGGAAGGAAAAG-3ʹ

Pt 2 Fragment R: 5ʹ-AACCAGGATCCGGATAAAAATGTGTAGCAGTAACAG-3ʹ

Pt 3, No. 3 Vector F: 5ʹ-TAGCGTTAACATCGATATCCGG-3ʹ

Pt 3, No. 3 Vector R: 5ʹ-GCTAACAAAGCACGGAGTTTAC-3ʹ

Pt 3, No. 3 Fragment F: 5ʹ-CCGTGCTTTGTTAGCTCCTCAGTCTTACGGAATC-3ʹ

Pt 3, No. 3 Fragment R: 5ʹ-TCGATGTTAACGCTATCCACCAGCAATCATCTTG-3ʹ

Primers used for mutagenesis

No. 1 F: 5ʹ-TGAGCAACGAGAGCCAGGCAAAAGAG-3ʹ

No. 1 R: 5ʹ-GGCTCTCGTTGCTCACCCTTCTCACC-3ʹ

No. 2 F: 5ʹ-GAGCAAGTAGAGCCAGGCAAAAGAGTA-3ʹ

No. 2 R: 5ʹ-TGGCTCTACTTGCTCACCCTTCTCAC-3ʹ

No. 3 F: 5ʹ-AGGAGATTAAGGACCACCAGGACTCC-3ʹ

No. 3 R: 5ʹ-GGTCCTTAATCTCCTTTCAAACCAGG-3ʹ
